# Pareto-optimal phylogenetic tree reconciliation

**DOI:** 10.1093/bioinformatics/btu289

**Published:** 2014-06-11

**Authors:** Ran Libeskind-Hadas, Yi-Chieh Wu, Mukul S. Bansal, Manolis Kellis

**Affiliations:** ^1^Department of Computer Science, Harvey Mudd College, Claremont, CA 91711, ^2^Department of Electrical Engineering and Computer Science, MIT, Cambridge, MA 02139, ^3^Department of Computer Science and Engineering, University of Connecticut, Storrs, CT 06269 and ^4^Broad Institute, Cambridge, MA 02142, USA

## Abstract

**Motivation:** Phylogenetic tree reconciliation is a widely used method for reconstructing the evolutionary histories of gene families and species, hosts and parasites and other dependent pairs of entities. Reconciliation is typically performed using maximum parsimony, in which each evolutionary event type is assigned a cost and the objective is to find a reconciliation of minimum total cost. It is generally understood that reconciliations are sensitive to event costs, but little is understood about the relationship between event costs and solutions. Moreover, choosing appropriate event costs is a notoriously difficult problem.

**Results:** We address this problem by giving an efficient algorithm for computing Pareto-optimal sets of reconciliations, thus providing the first systematic method for understanding the relationship between event costs and reconciliations. This, in turn, results in new techniques for computing event support values and, for cophylogenetic analyses, performing robust statistical tests. We provide new software tools and demonstrate their use on a number of datasets from evolutionary genomic and cophylogenetic studies.

**Availability and implementation:** Our Python tools are freely available at www.cs.hmc.edu/∼hadas/xscape.

**Contact:** mukul@engr.uconn.edu

**Supplementary information:** Supplementary data are available at *Bioinformatics* online.

## 1 INTRODUCTION

Phylogenetic tree reconciliation is a fundamental technique for studying the evolution of pairs of entities such as gene families and species, parasites and hosts and species and geographical regions. Recent algorithmic advances in tree reconciliation have led to seminal biological discoveries. Among these are the finding that >26% of extant gene families arose during the Archaean period between 3.33 and 2.85 billion years ago (David and Alm, 2011), a study showing that interactions of species and their ecological niches are strongly conserved across the entire tree of life (Gómez *et al.*, 2010), and new insights into the relationship between pathogenic RNA viruses and their hosts (Jackson and Charleston, 2004).

The reconciliation problem takes as input two trees and the associations between their leaves and seeks a mapping of one tree onto the other such that incongruence between the two trees is accounted for by a set of evolutionary events. In the context of gene family evolution, the two trees are the *gene tree* and the *species tree*, and in the well-studied Duplication-Transfer-Loss model (DTL), the events are speciation, duplication, transfer and loss. Unlike the simpler Duplication-Loss (DL) reconciliation model (Goodman *et al.*, 1979: Page, 1994), DTL accounts for transfer events and is thus broadly applicable across the tree of life. In the context of parasites and their hosts, the corresponding events are co-speciation, independent speciation, host switch and loss, respectively. In the context of species and area cladograms, these four events correspond to vicariance, sympatric speciation, dispersal and loss, respectively (Morrone, 2009). Henceforth, we refer to this set of events as the DTL model and use the DTL event names.

DTL-reconciliation is generally performed in a maximum parsimony framework in which each event type has an associated user-defined cost and the objective is to find a reconciliation of minimum total cost. Probabilistic approaches for DTL reconciliation, which do not require event cost assignments, also exist (Szollosi *et al.*, 2012; Tofigh, 2009), but these require estimates of other parameters, such as species divergence times, and are prohibitively slow for trees with more than a few leaves. If the species trees are fully dated, then maximum parsimony reconciliations can be found in polynomial time (Doyon *et al.*, 2010; Libeskind-Hadas and Charleston, 2009). However, accurately dating the internal nodes of a phylogenetic tree is generally difficult (Rutschmann, 2006). In the absence of dates, reconciliations may be time-inconsistent in the sense that they can induce contradictory constraints on the relative order of the internal nodes. The problem of finding optimal time-consistent DTL-reconciliations in undated trees is known to be NP-hard (Hallett *et al.*, 2004; Ovadia *et al.*, 2011). Therefore, a common approach, and the one followed in this article, is to relax the time-consistency requirement (Bansal *et al.*, 2012, 2013; Chen *et al.*, 2012; David and Alm, 2011; Tofigh *et al.*, 2011), which permits an optimal (although not necessarily time-consistent) solution to be found in *O*(*mn*) time (Bansal *et al.*, 2012), where *m* and *n* denote the number of nodes in the gene (parasite) and species (host) trees, respectively. Experimental evidence suggests that the solutions found using this approach are generally time-consistent (Addario-Berry *et al.*, 2003); but see also Stolzer *et al.* (2012).

**Fig. 1.**
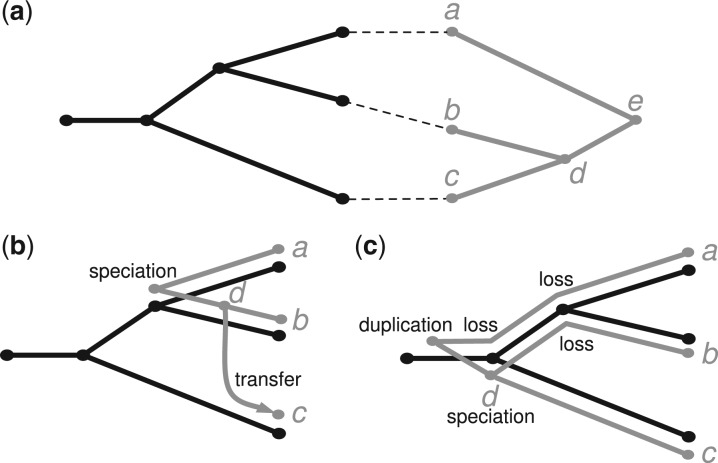
(**a**) A species (host) tree in black and a gene (parasite) tree in gray with the leaf associations shown in dotted lines. (**b** and **c**) Two different reconciliations with events labeled by type

Maximum parsimony reconciliations depend on the event costs. In the DTL model, speciations are considered ‘null events’ and are therefore typically assigned a cost of 0 while duplications, transfers and losses are assigned positive costs. Figure 1a shows a species (host) tree in black and a gene (parasite) tree in gray along with associations between their leaves. If duplication, transfer and loss each cost 1, then the reconciliation in Figure 1b is optimal, comprises one speciation and one transfer and has total cost 1. However, if duplication and loss cost 1 and transfer costs 5, then the maximum parsimony reconciliation in Figure 1c is optimal, comprises one speciation, one duplication and three losses and has total cost 4. Even small differences in event costs can induce different solutions when the trees are larger. For example, we have found that in many datasets, the default costs used in TreeMap (Charleston, 1998) and Jane (Conow *et al.*, 2010) give rise to different reconciliations than those using the default costs in AnGST (David and Alm, 2011) and RANGER-DTL (Bansal *et al.*, 2012).

Despite great advances in the efficiency and accuracy of DTL-reconciliation, little is understood about the relationship between event costs and the resulting maximum parsimony reconciliations. A systematic way to handle the difficulty in determining appropriate event costs is to optimize for event counts rather than total numerical cost. We define an *event count vector* for a reconciliation to be a triple 〈δ,θ,ℓ〉, denoting the number of duplications, transfers and losses, respectively. These vectors do not explicitly count the number of speciation events because the number of speciations is implicit (it is m−δ−θ−1).

An event count vector v=〈δ,θ,ℓ〉 is *strictly better* than an event count vector v′=〈δ′,θ′,ℓ′〉 if each entry of *v* is less than or equal to the corresponding entry in *v^′^* and at least one entry of *v* is less than its corresponding entry in *v^′^*. A reconciliation is *Pareto-optimal* if there is no reconciliation with a strictly better event count vector. We use the term Pareto-optimal for both reconciliations and their corresponding event count vectors.

Given the set of all Pareto-optimal event count vectors, we can partition the space of possible event cost assignments into equivalence classes, or ‘regions’, such that any two event cost assignments within the same region lead to the same optimal reconciliations. These regions provide insights into the relationships between event costs and maximum parsimony reconciliations and have numerous applications, including new definitions and algorithms for computing event consensus support.

### Previous work

TreeMap (Charleston, 1998) was the first to address the problem of uncertain event costs by enumerating Pareto-optimal solutions. However, TreeMap’s underlying algorithm has worst-case exponential time and thus can only be used with small trees. Tofigh (2009) later considered the problem of computing all Pareto-optimal solutions for the DTL-reconciliation problem but without accounting for losses; this simplifies the algorithmic problem but affects the accuracy of the reconciliation. Losses play a fundamental role in the ability to distinguish between duplications and transfers, and in mapping the nodes of the gene tree to the nodes of the species tree, and thus should be explicitly considered during reconciliation (Stolzer *et al.*, 2012).

Thus, despite earlier work on Pareto-optimal reconciliations, there currently exist no algorithms or tools for computing or exploring the space of all Pareto-optimal DTL reconciliations.

### Our contributions

In this article, we give efficient algorithms that compute all Pareto-optimal DTL reconciliations and completely characterize the relationship between event costs and maximum parsimony reconciliations. Specifically,

- We give an algorithm for computing the Pareto-optimal event count vectors by extending the dynamic programming approaches that we and others have developed for the case of fixed event costs. Our algorithm has worst-case running time $O(m^{5}n\log m)$ where *m* and *n* denote the number of leaves in the gene (parasite) tree and species (host) tree, respectively. The algorithm also counts the number of distinct reconciliations associated with each event count vector. In addition, we give a $O(m^{4}\log m)$-time algorithm that uses the Pareto-optimal event count vectors to partition the event cost space into equivalence classes, or regions, such that event costs in a region give rise to the same set of maximum parsimony reconciliations.
- We present three applications of this algorithm and provide downloadable software tools for each one.
  – The first tool, *costscape*, computes the Pareto-optimal event count vectors and provides a visualization of the corresponding regions.
  – The second tool, *eventscape*, identifies the individual events that are common to the reconciliations in each region and uses this information to identify events that are strongly supported across the event cost space.
  – The *sigscape* tool permits new, more robust statistical significance tests in cophylogenetic analyses.
- We apply these tools to a number of datasets to demonstrate their utility. These results show that a small number of appropriately selected event costs can be used to capture a large fraction of maximum parsimony reconciliations; that a significant fraction of speciation events occur in every reconciliation across a wide range of event costs; and that duplications, transfers and losses are more sensitive to the choice of event costs.

## 2 DEFINITIONS AND PRELIMINARIES

We follow the basic definitions and notation from Bansal *et al.* (2012). Given a tree *T*, we denote its node, edge and leaf sets by V(T),E(T) and Le(T), respectively. If *T* is rooted, the root node of *T* is denoted by rt(T), the parent of a node v∈V(T) by $pa_{T}(v)$, its set of children by $Ch_{T}(v)$ and the (maximal) subtree of *T* rooted at *v* by *T*(*v*). The set of *internal nodes* of *T*, denoted *I*(*T*), is defined to be V(T)∖Le(T). We define $\leq_{T}$ to be the partial order on *V*(*T*) where $x\leq_{T}y$ if *y* is a node on the path between rt(T) and *x*. The partial order $\geq_{T}$ is defined analogously, i.e. $x\geq_{T}y$ if *x* is a node on the path between rt(T) and *y*. We say that *y* is an *ancestor* of *x*, or that *x* is a *descendant* of *y*, if $x\leq_{T}y$ (note that, under this definition, every node is a descendant as well as ancestor of itself). We say that *x* and *y* are *incomparable* if neither $x\leq_{T}y$ nor $y\leq_{T}x$. Given a non-empty subset L⊆Le(T), we denote by $lca_{T}(L)$ the last common ancestor (LCA) of all the leaves in *L* in tree *T*, that is, $lca_{T}(L)$ is the unique smallest upper bound of *L* under $\leq_{T}$. Given $x,y\in V(T),x\to_{T}y$ denotes the unique path from *x* to *y* in *T*. We denote by $d_{T}(x,y)$ the number of edges on the path $x\to_{T}y$; note that if *x* = *y* then $d_{T}(x,y)=0$. Throughout this work, the *term* tree refers to a rooted binary tree.

We assume that the two input trees are denoted by *T* and *S*, and the goal is to map tree *T* to tree *S*. Thus, in gene-tree/species-tree reconciliation, *T* denotes the gene tree and *S* the species tree; in coevolutionary studies, *T* denotes the parasite tree and *S* the host tree; and in biogeographical studies, *T* denotes the species tree and *S* the area cladogram. Each leaf of tree *T* is labeled with the leaf-label from *S* with which it is associated. This labeling defines a *leaf-mapping* $L_{T,S}:Le(T)\to Le(S)$ that maps a leaf node t∈Le(T) to the unique leaf node s∈Le(S), which has the same label as *t*. Note that *T* may have more than one leaf associated with the same leaf of *S*. Throughout this work we will implicitly assume that the species tree contains all the species represented in the gene tree.

### 2.1 Reconciliation and DTL scenarios

Next, we define what constitutes a valid DTL reconciliation; specifically, we define a Duplication-Transfer-Loss scenario (DTL-scenario) (Bansal *et al.*, 2012; Tofigh *et al.*, 2011) for *T* and *S* that characterizes the mappings of *T* into *S* that constitute a biologically valid reconciliation. Essentially, DTL-scenarios (i) map each node of *T* to a unique node in *S* in a consistent way that respects the immediate temporal constraints implied by the topology of *S*, and (ii) designate each node of *T* as representing either a speciation, duplication or transfer event.

**Definition 2.1 (DTL-scenario)**

*A DTL-scenario for T and S is a seven-tuple* 〈L,M,Σ,Δ,Θ,Ξ,τ〉*, where* L:Le(T)→Le(S) *represents the leaf-mapping from T to* S,M:V(T)→V(S) *maps each node of T to a node of S, the sets* Σ,Δ *and Θ partition I(T) into speciation (or co-speciation), duplication and transfer nodes, respectively, Ξ is a subset of edges of T that represent transfer edges and* τ:Θ→V(S) *specifies the recipient for each transfer event, subject to the following constraints:*

1. *If* t∈Le(T)*, then* M(t)=L(t)*.*
2. *If* t∈I(T) *and t′ and t″ denote the children of t, then,*
  - M(t)≮M(t′) *and *M(t)≮M(t″)*,*
  - *At least one of *M(t′) *and *M(t″) *is a descendant of *M(t)*.*
3. *Given any edge *(t,t′)∈E(T),(t,t′)∈Ξ *if and only if *M(t) *and *M(t′) *are incomparable.*
4. *If *t∈I(T) *and *t′ *and *t″ *denote the children of t, then,*
  - t∈Σ *only if *M(t)=lca(M(t′),M(t″)) *and *M(t′) *and *M(t″) *are incomparable,*
  - t∈Δ *only if *$M(t)\geq_{S}\;lca(M(t\prime ),M(t\prime \prime ))$*,*
  - t∈Θ *if and only if either *(t,t′)∈Ξ *or *(t,t″)∈Ξ*.*
  - *If *t∈Θ *and *(t,t′)∈Ξ*, then *M(t) *and *τ(t) *must be incomparable, and *M(t′) *must be a descendant of *τ(t)*.*

Constraint 1 above ensures that the mapping M is consistent with the leaf-mapping L. Constraint 2a imposes on M the temporal constraints implied by *S*. Constraint 2b implies that any internal node in *G* may represent at most one transfer event. Constraint 3 determines the edges of *T* that are transfer edges. Constraints 4a, 4b and 4c state the conditions under which an internal node of *T* may represent a speciation, duplication and transfer, respectively. Constraint 4d specifies which species may be designated as the recipient species for any given transfer event.

DTL-scenarios correspond naturally to reconciliations, and it is straightforward to infer the reconciliation of *T* and *S* implied by any DTL-scenario.

Given a DTL-scenario, one can directly count the minimum number of gene losses (Bansal *et al.*, 2012) in the corresponding reconciliation as follows.

**Definition 2.2 (Losses)**

*Given a DTL-scenario *α=〈L,M,Σ,Δ,Θ,Ξ,τ〉 *for T and S, let *t∈V(T) *and *{t′,t″}=Ch(t)*. The number of losses *$Loss_{\alpha }(t)$ *at node t is defined to be*

- $\left|d_{S}(M(t),M(t\prime ))-1|+|d_{S}(M(t),M(t\prime \prime ))-1\right|$, if t∈Σ.
- $d_{S}(M(t),M(t\prime ))+d_{S}(M(t),M(t\prime \prime ))$, if t∈Δ.
- $d_{S}(M(t),M(t\prime \prime ))+d_{S}(\tau (t),M(t\prime ))$, if (t,t′)∈Ξ.

*The total number of losses in the reconciliation corresponding to the DTL-scenario α is defined to be* 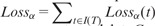.

We assume that speciations have zero cost, and let $C_{\Delta },C_{\Theta }$ and *C_L_* denote the assigned positive costs for duplication, transfer and loss events, respectively. Then the cost of reconciling *T* and *S* according to a DTL-scenario α is defined as follows:

**Definition 2.3 (Reconciliation cost of a DTL-scenario)**

*Given a DTL-scenario *α=〈L,M,Σ,Δ,Θ,Ξ,τ〉 *for T and S, the reconciliation cost associated with α is given by *$C_{\Delta }\cdot |\Delta |+C_{\Theta }\cdot |\Theta |+C_{L}\cdot Loss_{\alpha }$*.*

The traditional goal of DTL-reconciliation is to find a most parsimonious reconciliation, i.e. a DTL-scenario for *G* and *S* with minimum reconciliation cost. However, in this work, we assume that the exact cost assignments, $C_{\Delta },C_{\Theta }$ and *C_L_*, are unknown; therefore, we focus on the inferred event counts rather than the reconciliation cost itself.

**Definition 2.4 (Event count vector)**

*Given a DTL-scenario *α=〈L,M,Σ,Δ,Θ,Ξ,τ〉 *for T and S, the event count vector associated with α, denoted *$v_{\alpha }$*, is defined to be *$\langle |\Delta |,|\Theta |,Loss_{\alpha }\rangle$*.*

Given two DTL-scenarios α=〈L,M,Σ,Δ,Θ,Ξ,τ〉 and α′=〈L,M,Σ′,Δ′,Θ′,Ξ′,τ′〉 for *T* and *S*, the event count vector $v_{\alpha }$ is said to be *strictly better* than $v_{\alpha \prime }$ if each entry of $v_{\alpha }$ is less than or equal to the corresponding entry in $v_{\alpha \prime }$ and at least one entry of $v_{\alpha }$ is less than its corresponding entry in $v_{\alpha \prime }$.

**Definition 2.5 (Pareto-optimal event count vector)**

*Given a DTL-scenario *α=〈L,M,Σ,Δ,Θ,Ξ,τ〉 *for T and S, the event count vector *$v_{\alpha }$ *is said to be Pareto-optimal if there does not exist any other DTL-scenario *α′ *for T and S whose event count vector *$v_{\alpha \prime }$ *is strictly better than *$v_{\alpha }$*.*

Given trees *T* and *S* and leaf-mapping $L_{T,S}$, our goal is to find all Pareto-optimal event count vectors for the two trees. Note that, if an event count vector 〈δ,θ,ℓ〉 is Pareto-optimal, then there exists some assignment of values for $C_{\Delta },C_{\Theta }$ and *C_L_* for which a most parsimonious reconciliation invokes exactly δ duplications, θ transfers and ℓ losses. Thus, the set of all Pareto-optimal event count vectors provides a relationship between event cost assignments and most parsimonious reconciliations.

**Problem 1 (Pareto-optimal vectors)**

*The Pareto-optimal vectors (PV) problem is to find the set of all Pareto-optimal event count vectors for trees T and S and their leaf-mapping *$L_{T,S}$*.*

The set of Pareto-optimal vectors obtained by solving the PV problem can then be used to partition the space of possible event cost assignments into ‘equivalent’ regions.

**Problem 2 (Equivalent Region Partition)**

*Given *$T,S,L_{T,S}$ *and the set of all Pareto-optimal event count vectors for the two trees, the equivalent region partition (ERP) problem is to partition the space of possible event cost assignments into disjoint regions such that any two event cost assignments within the same region yield the same set of maximum parsimony reconciliations.*

In the next section, we first show how to efficiently solve both the PV and ERP problems. In the subsequent sections, we use these algorithmic results as the basis of new software tools and then demonstrate the utility of these tools on several biological datasets.

## 3 ALGORITHMS

Our algorithm for the PV problem is based on an extension of the dynamic programming framework for computing most parsimonious DTL reconciliations with known event costs, e.g. (Bansal *et al.*, 2012). Tofigh (2009) was the first to adapt the dynamic programming algorithm to compute Pareto-optimal event count vectors. However, that result did not count losses, which significantly simplifies the dynamic programming algorithm and also bounds the number of Pareto-optimal event count vectors to just *O*(*m*), resulting in an $O(m^{3}n)$-time algorithm, where m=|Le(T)| and n=|Le(S)|. In developing an efficient algorithm for the PV problem, we not only show how to efficiently account for losses in the dynamic programming algorithm [using ideas from Bansal *et al.* (2012)] but also show how to efficiently maintain the resulting larger set of Pareto-optimal event count vectors.

### 3.1 Solving the PV problem

Given any t∈I(T) and s∈V(S), let $P_{\Sigma }(t,s)$ denote the set of Pareto-optimal event count vectors for reconciling *T*(*t*) with *S* such that *t* maps to *s* and t∈Σ. The terms $P_{\Delta }(t,s)$ and $P_{\Theta }(t,s)$ are defined similarly for t∈Δ and t∈Θ, respectively. Given any t∈V(T) and s∈V(S), we define P(t,s) to be the set of Pareto-optimal event count vectors for reconciling *T*(*t*) with *S* such that *t* maps to *s*. Our algorithm performs a nested post-order traversal of *T* and *S* to compute P(t,s) for each *t* and *s*.

Given two sets *A* and *B* of Pareto-optimal event count vectors, we define A⊕B to be the set obtained by taking the union of the event count vectors in *A* and *B* and then selecting the subset of event count vectors that are Pareto-optimal. Similarly, A⊗B is defined to be the set obtained by first computing the Cartesian product of *A* and *B*, then converting each resulting ordered pair into a single event count vector by adding the two vectors of the ordered pair, and finally taking only the subset of Pareto-optimal event count vectors. These operations will be used when merging the Pareto-optimal event count vectors from smaller subproblems to compute the Pareto-optimal event count vectors for larger subproblems. The dynamic programming table for P(t,s) is initialized and computed as shown below:

$$P(t,s)=\{\begin{array}{c} \langle 0,0,0\rangle \text{ if}\;t\in Le(T)\;\text{and}\;s=M(t),\\ \langle \infty ,\infty ,\infty \rangle \;\;\text{if}\;t\in Le(T)\;\text{and}\;s\neq M(t),\\ P_{\Sigma }(t,s)⊕P_{\Delta }(t,s)⊕P_{\Theta }(t,s)\;\;\text{otherwise}. \end{array}$$

Given an event count vector, v=〈δ,θ,ℓ〉, we use the notation v+(Δ,i), for $i\in {\mathbb{Z}}^{+}$, to denote the event count vector where the count for duplications is incremented by *i*, i.e. 〈δ+i,θ,ℓ〉. The vectors v+(Θ,i) and v+(L,i) are defined analogously for transfers and losses, respectively. We extend this notation to a set of event count vectors, *A*, as follows: A+(Δ,i) represents the set {v+(Δ,i):v∈A}. The sets A+(Θ,i) and A+(L,i) are defined analogously. We define

$in(t,s)=\underset{x\in V(S(s))}{⊕}(P(t,x)+(L,d_{S}(s,x)))$,
$out(t,s)=\underset{x\in V(S)\;\text{incomparable to}\;s}{⊕}P(t,x)$, and
$inAlt(t,s)=\underset{x\in V(S(s))}{⊕}P(t,x)$.

Our algorithm computes $P_{\Sigma }(t,s),P_{\Delta }(t,s)$ and $P_{\Theta }(t,s)$ for each t∈V(T) and s∈V(S) by performing a nested post-order traversal of *T* and *S*. The values in(·,·),out(·,·) and inAlt(·,·) help to reuse previously computed information to efficiently compute the values $P_{\Sigma }(t,s),P_{\Delta }(t,s)$ and $P_{\Theta }(t,s)$ at each step. The exact formulas for computing the values of $P_{\Sigma }(t,s),P_{\Delta }(t,s)$ and $P_{\Theta }(t,s)$ using previously computed values are given in steps 17, 18 and 19 of the algorithm below. The nested post-order traversal ensures that when computing $P_{\Sigma }(t,s),P_{\Delta }(t,s)$ and $P_{\Theta }(t,s)$ at nodes t∈G and s∈S, all the required in(·,·),out(·,·),inAlt(·,·) and P(·,·) values have already been computed.

Note that once all the P(·,·) sets have been computed, the set of Pareto-optimal event count vectors for the reconciliation of *T* and *S* is simply $\underset{s\in V(S)}{⊕}P(rt(T),s)$. The algorithm is as follows:

**Algorithm** Pareto−Reconcile(T,S,L)

1: **for** each t∈V(T) and s∈V(S) **do**

2: Initialize $P(t,s),P_{\Sigma }(t,s),P_{\Delta }(t,s),P_{\Theta }(t,s),in(t,s),inAlt(t,s)$, and out(t,s) to ∅.

3: **for** each t∈Le(T) **do**

4:  P(t,L(t))={〈0,0,0〉}, and, for each $s\geq_{S}L(t)$, $in(t,s)=\{\langle 0,0,d_{S}(s,L(t))\rangle \}$ and inAlt(t,s)={〈0,0,0〉}.

5: **for** each t∈I(T) in post-order **do**

6:  **for** each s∈V(S) in post-order **do**

7:   Let $\left\{t\prime ,t\prime \prime \}=Ch_{T}(t\right)$.

8:   **if** s∈Le(S) **then**

9:    $P_{\Sigma }(t,s)=\{\langle \infty ,\infty ,\infty \rangle \}$.

10:    $P_{\Delta }(t,s)=(P(t\prime ,s)⊗P(t\prime \prime ,s))+(\Delta ,1)$.

11:    $P_{\Theta }(t,s)=((in(t\prime ,s)⊗out(t\prime \prime ,s))⊕$

          (in(t″,s)⊗out(t′,s)))+(Θ,1).

12:    $P(t,s)=P_{\Sigma }(t,s)⊕P_{\Delta }(t,s)⊕P_{\Theta }(t,s)$.

13:    in(t,s)=P(t,s).

14:    inAlt(t,s)=P(t,s).

15:  **else**

16:    Let $\left\{s\prime ,s\prime \prime \}=Ch_{S}(s\right)$.

17:    $P_{\Sigma }(t,s)=(in(t\prime ,s\prime )⊗in(t\prime \prime ,s\prime \prime ))⊕$

          (in(t″,s′)⊗in(t′,s″)).

18:    $P_{\Delta }(t,s)=(in(t\prime ,s)⊗in(t\prime \prime ,s))+(\Delta ,1)$.

19:    If s≠rt(S), then $P_{\Theta }(t,s)=((in(t\prime ,s)⊗out(t\prime \prime ,s))⊕$

         (in(t″,s)⊗out(t′,s)))+(Θ,1).

20:    $P(t,s)=P_{\Sigma }(t,s)⊕P_{\Delta }(t,s)⊕P_{\Theta }(t,s)$.

21:    in(t,s)=P(t,s)⊕(in(t,s′)+(L,1))⊕

         (in(t,s″)+(L,1)).

22:    inAlt(t,s)=P(t,s)⊕inAlt(t,s′)⊕inAlt(t,s″).

23:  **for** each s∈I(S) in pre-order **do**

24:    Let $\left\{s\prime ,s\prime \prime \}=Ch_{S}(s\right)$.

25:    out(t,s′)=out(t,s)⊕inAlt(t,s″), and

     out(t,s″)=out(t,s)⊕inAlt(t,s′).

26: Return $\underset{s\in V(S)}{⊕}P(rt(T),s)$.

To complete our description of the above algorithm, we must also show how to efficiently perform the operations A⊕B and A⊗B, for any two sets *A* and *B* of Pareto-optimal event count vectors. Operation A⊕B can be performed in $O(m^{4})$ time using a straightforward algorithm (Lemma 3.2 below). Computing A⊗B efficiently is more involved. From Lemma 3.1 (below), each Pareto-optimal set has size $O(m^{2})$. Thus, computing their Cartesian product takes time $O(m^{4})$. Finding the subset of Pareto-optimal vectors by pairwise comparisons would thus take $O({\left(m^{2}\times m^{2}\right)}^{2})=O(m^{8})$ time. We now show how A⊗B can be computed in $O(m^{4}\log m)$ time.

**Procedure** Perform−⊗(A,B)

1: Create an empty ordered list *Z* that will be used to store event count vectors in lexicographically sorted order.

2: **for** each a∈A **do**

3:  **for** each b∈B **do**

4:   Compute the vector a+b. Let this new vector be denoted as c=〈δ,θ,ℓ〉.

5:   Insert *c* into *Z* (maintaining lexicographic order).

6:   Consider the element *d* immediately before *c* in the list *Z*.

7:   **if** *d* exists and is of the form 〈δ,θ,ℓ−x〉, where x≥0    **then**

8:    Delete *c* from *Z*.

9:   **else**

10:    Consider the element *e* immediately after *c* in the list *Z*.

11:    **if** *e* exists and is of the form 〈δ,θ,ℓ+x〉, where x≥0     **then**

12:     Delete *e* from *Z*.

13: Delete from *Z* all vectors that are not Pareto-optimal.

14: Return *Z*.

We now analyze our algorithm and prove its correctness. Recall that *m* and *n* denote the number of leaves in *T* and *S*, respectively.

**Lemma 3.1**

*The cardinality of any set of Pareto-optimal event count vectors can be no greater than *$\Theta (m^{2})$*.*

**Proof**

Consider any event count vector 〈δ,θ,ℓ〉. Let *A* be any set of Pareto-optimal event count vectors. Suppose *A* contains more than one vector with identical values for δ and θ but different values for *ℓ*. Clearly, only one of these vectors can be Pareto-optimal (the one with the lowest value for *ℓ*). Thus, for any pair of fixed values for δ and θ, *A* may contain at most one vector with that assignment of values for δ and θ. Because the values of δ and θ are both bounded by *m* − 1, the number of internal nodes in *T*, the lemma follows.

**Lemma 3.2**

*Given any two sets, A and B, of Pareto-optimal event count vectors, the set *A⊕B *can be computed in *$O(m^{4})$ *time.*

**Proof**

Let C=A⊕B. Because both *A* and *B* have $O(m^{2})$ elements, so does *C*. Thus, trimming down set *C* to just the Pareto-optimal vectors requires at most $O(m^{4})$ time.

**Lemma 3.3**

*Given any two sets, A and B, of Pareto-optimal event count vectors, the set *A⊗B *can be computed in *$O(m^{4}\log m)$ *time.*

**Proof**

Consider Procedure Perform−⊗(A,B). We first prove its correctness and then analyze its time complexity.

*Correctness:* Consider the set $Y=\{v_{1}+v_{2}:v_{1}\in A\;\text{and}\;v_{2}\in B\}$. The procedure constructs each element of *C*, one at a time, and adds it to the ordered set *Z*. The only elements ever deleted from set *Z* in Steps 8 and 12 are those that are not Pareto-optimal. Finally, Step 13 removes any remaining elements that are not Pateto-optimal from *Z*. The procedure thus computes the value of A⊗B correctly.

*Complexity:* Because both *A* and *B* contain at most $O(m^{2})$ vectors (Lemma 3.1), Steps 4 through 12 are each executed $O(m^{4})$ times. By using a self-balancing binary search tree to represent *Z*, each of these steps can be executed in O(log⁡m) time, yielding a total time complexity of $O(m^{4}\log m)$ for Steps 1 through 12. Because the time complexity of Step 13 is $O(|Z{|}^{2})$, it now suffices to show that the size of *Z* never exceeds *m*^2^ at any time. Consider Steps 8 and 12. These steps ensure that for any fixed value of δ, and θ, there is at most one vector in *Z* of the form 〈δ,θ,ℓ〉. Thus, the size of *Z* can not exceed $O(m^{2})$ at any time.

Based on the pseudo-code for Algorithm *Pareto-Reconcile*, and on the previous three lemmas, the next two theorems follow easily. For brevity, their proofs appear in Supplementary Section S1.

**Theorem 3.1**

Algorithm Pareto-Reconcile correctly solves the PV problem.

**Theorem 3.2**

*The total time complexity of Algorithm Pareto-Reconcile is *$O(m^{5}n\log m)$*. In addition, the algorithm can be implemented so that its total space complexity is *$O(m^{3}n)$*.*

### 3.2 Equivalent region partition

In this section, we describe how the set of Pareto-optimal event count vectors can be used to efficiently partition the space of event costs into a finite number of equivalence classes, or ‘regions’, such that all event costs in a given region induce the same set of maximum parsimony reconciliations. These regions provide insights into the relationship between the event costs and the resulting solutions and are used in several of the software tools described in the next section.

Recall that we assume that speciation is a ‘null’ event with cost 0 and all other events have positive costs. Because the costs are unit-less, duplication cost is normalized to 1 and the costs of transfer and loss are non-negative values relative to the unit cost of duplication. For a given event count vector v=〈δ,θ,ℓ〉 and positive real transfer and loss costs $C_{\Theta }$ and $C_{\ell }$, respectively, the cost of that solution, denoted $C(v,C_{\Theta },C_{\ell })$, is $\delta +C_{\Theta }\cdot \theta +C_{L}\cdot \ell$.

Let *A* denote the Pareto-optimal set of event count vectors for a given pair of trees and leaf mapping. These vectors induce a partition of the event cost space into *regions* where the region *R*(*v*) associated with event count vector v∈A is the set of points $(C_{\theta },C_{\ell })\in {\mathbb{R}}^{+}\times {\mathbb{R}}^{+}$ such that $C(v,C_{\Theta },C_{\ell })\leq C(v\prime ,C_{\Theta },C_{\ell }),\forall v\prime \in A-v$.

From this definition, it follows that for every combination of transfer and loss costs in a given region, every maximum parsimony solution using those costs will have the event count vector associated with that region. While there can be many distinct reconciliations in a given region, all event costs in that region will admit the same set of maximum parsimony reconciliations.

**Theorem 3.3**

*Given a set of Pareto-optimal event count vectors, the corresponding regions can be found in time *$O(m^{4}\log m)$*.*

**Proof**

Let *A* denote the set of Pareto-optimal event count vectors. By Lemma 3.1, $\left|A|\in O(m^{2}\right)$. The region *R*(*v*) corresponding to v∈A comprises all points $(C_{\theta },C_{\ell })\in {\mathbb{R}}^{+}\times {\mathbb{R}}^{+}$ such that $C(v,C_{\Theta },C_{\ell })\leq C(v\prime ,C_{\Theta },C_{\ell }),\forall v\prime \in A-v$. Each inequality of the form $C(v,C_{\Theta },C_{\ell })\leq C(v\prime ,C_{\Theta },C_{\ell })$ induces a half-space and *R*(*v*) is the intersection of those half-spaces. The intersection of *N* half-spaces can be found in time O(Nlog⁡N) (Berg *et al.*, 2008) and thus each region can be found in time $O(m^{2}\log m)$. Thus, all |A| regions can be found in time $O(m^{4}\log m)$.

### 3.3 Counting solutions and enumerating events

Our algorithm for the PV problem can be easily adapted to count the number of maximum parsimony reconciliations for each Pareto-optimal event count vector and to record the events in those reconciliations. To count the number of distinct reconciliations, we keep track of the number of solutions associated with each event count vector in each subproblem P(t,s). Those values are easily updated based on the number of solutions from the subproblems from which P(t,s) is constructed (Bansal *et al.*, 2013). The additional bookkeeping does not increase the asymptotic running time of the algorithm.

In addition, the dynamic program can be augmented to keep track of the set of events occurring in the reconciliations associated with a Pareto-optimal event count vector. While the number of reconciliations can grow exponentially with the *m* and *n*, the total number of distinct events is bounded by $O(mn^{2})$ because each of the *O*(*m*) nodes of *T* can be mapped to at most *O*(*n*) nodes in *S* and, for transfer events, there are *O*(*n*) possible destinations for the ‘landing site’. For each subproblem P(t,s) we can, therefore, maintain a set of associated events such as the union or intersection of all events that occur in that subproblem. This increases the asymptotic running time by a factor that depends only on the time complexity of the particular set theoretic operation. For example, we use set intersection in the *eventscape* tool described in the next section, which contributes a multiplicative factor of $O(mn^{2}\log mn)$ using self-balancing binary search trees.

## 4 APPLICATIONS AND SOFTWARE

In this section, we demonstrate three programs that use the algorithmic results in the previous section to provide new insights into maximum parsimony reconciliation. Each of these tools solves the PV and ERP problems and uses those solutions in different ways. We note that while our algorithms can compute all Pareto-optimal regions, these tools take a user-specified a range of costs for transfer and loss events, relative to the normalized unit cost of duplication, and restrict the Pareto-optimal event count vectors and corresponding regions to that *event cost space*. (We note that the choice of fixing the duplication cost to 1 and normalizing the remaining costs with respect to duplication is arbitrary).

**Fig. 2.**
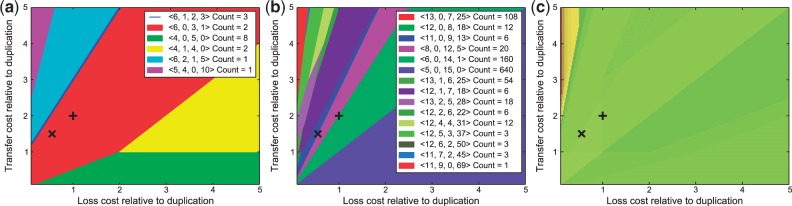
Pareto-regions for the (**a**) gopher-louse and (**b**) indigobird-finch datasets. Colors are arbitrary and are used to match regions with the event counts in the legend. The displayed event counts comprise the number of speciations in addition to the number of duplications, transfers and losses. In addition, the ‘Count’ field indicates the number of distinct reconciliations in each region. While most regions are polygons, regions may also be points or lines, such as in the first region in the legend in (a). (**c**) Results of a permutation test on the gopher-louse dataset using 1000 random trials. For (c) only, green indicates significance at the 0.01 level, yellow represents significance between 0.01 and 0.05 and red indicates lack of significance at the 0.05 level. Brightness of green, yellow and red indicates differences in *P*-values, with brighter shades indicating smaller *P*-values. All plots use transfer and loss costs ranging from 0.1 to 5. Plus sign marks the default event costs for Jane/TreeMap ($C_{\Delta }=1,C_{\Theta }=2,C_{L}=1$) and multiplication sign the default event costs for AnGST/RANGER-DTL ($C_{\Delta }=1,C_{\Theta }=3/2,C_{L}=1/2$). In (a), the two sets of default costs are in the same region, whereas in (b) and in almost all but the smallest datasets, the two sets of default costs are in different regions

The first tool, *costscape*, computes the Pareto-optimal event count vectors and their corresponding regions as well as a ‘Count’ of the number of distinct maximum parsimony reconciliations in each region. These results are displayed graphically to provide a systematic overview of the relationship between event costs and the structure of the maximum parsimony solution space. For example, Figure 2a and b show the results of using costscape on the canonical gopher-louse (Hafner and Nadler, 1988) and indigobird-finch (Sorenson *et al.*, 2004) datasets for transfer and loss costs ranging from 0.1 to 5, relative to the unit cost of duplication.

The second tool, *eventscape*, augments the dynamic programming algorithm to compute the set of events that are common to *every* reconciliation in a region. Eventscape then collects all of these events over all regions and partitions that set into the set of events that are found in exactly one region, exactly two regions and so forth up to the total number of regions. Each event includes a gene (parasite) node, its association with a species (host) node and the type of event. In the case of transfers, the landing site of the event is also specified. One important application of eventscape is in identifying events that are highly supported by merit of occurring in a large fraction of the regions or the cost space. The next section explores this application on a large collection of datasets.

The third tool, *sigscape* is designed for cophylogenetic analyses where permutation tests are performed to test the null hypothesis that the host and parasite trees are similar owing to chance. Shades of each color indicate variations in *P*-values, with brighter shades indicating smaller *P*-values. For example, for the gopher-louse dataset using transfer and loss costs ranging from 0.1 to 5 and using 1000 permutations, sigscape found 97.6% of the event cost space to have significance at the 0.01 level, 1.5% to have significance between 0.01 and 0.05, and 0.9% below the 0.05 level (Fig. 2c). More details about sigscape are provided in Supplementary Section S2.

These tools, collectively called *xscape*, are written in Python and are freely available at www.cs.hmc.edu/∼hadas/xscape. As noted earlier, the underlying algorithms do not guarantee time-consistent solutions because finding such solutions is NP-hard (Ovadia *et al.*, 2011). However, if the species trees are fully dated, then our algorithms can be modified (resulting in slower polynomial-time algorithms) to guarantee time-consistency (Conow *et al.*, 2010).

## 5 RESULTS

In this section, we demonstrate the utility of our algorithms and tools on a diverse collection of datasets. Currently, reconciliation analyses infer evolutionary events by choosing a set of event costs (often the default costs in the software, although most tools recommend experimenting with different costs) and constructing reconciliations based on these costs. Because event costs are not easily estimated and the choice of costs affects the reconciliations, we seek here to understand the impact of event costs on the resulting reconciliations.

We first analyzed a biological dataset consisting of predominantly prokaryotic species sampled broadly from across the tree of life (David and Alm, 2011). This consisted of 4860 gene trees (with at least two extant genes) over 100 species, but, for efficiency, we restricted our analyses to a subset of 3433 gene families from 20 randomly sampled species and evaluated event counts and their corresponding event cost regions for transfer and loss costs ranging from 0.5 to 2 (with respect to the unit cost of duplication). The results presented here exclude 34 (<1.0%) gene families for which *eventscape* used more than the allocated 5 GB of RAM in our experimental setup.

**Fig. 3.**
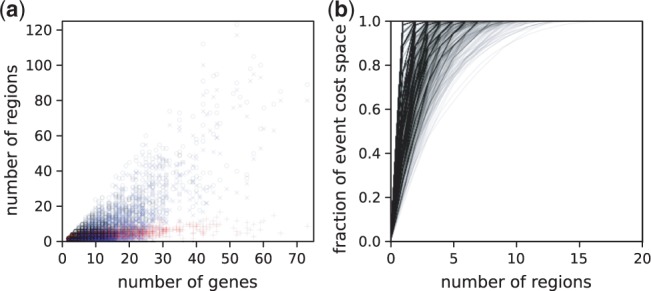
The *costscape* summary on the tree of life dataset. For each gene family, we computed (**a**) the number *y* of Pareto-optimal regions (all regions, black white circles; positive area regions, red plus sign; zero area regions, blue multiplication sign) and the number *x* of extant genes, and (**b**) the fraction *y* of the event cost space covered by the largest *x* regions

Using the *costscape* tool, we observed that 85.8% (2917) of the gene families induce at least two regions, 37.5% (1274) have at least five regions and that the number of regions grows as a function of tree size (Fig. 3a). Thus, the common practice of selecting a single cost setting or a small fixed number of cost settings [e.g., Stolzer *et al.* (2012) considered three settings, and Bansal *et al.* (2013) considered five] can result in missing potentially important parts of the solution space. Additionally, many regions have zero area (e.g. lines or points), meaning that the associated reconciliations are unlikely to be discovered by an ad hoc choice of event costs: for this dataset, 54.1% (1839) of the gene families have at least one region with zero area, and for 19.7% (669) of the gene families, more than half of the regions have zero area.

We also found that a systematic choice of event costs (e.g. using *costscape*) can cover a substantial portion of the solution space. For example, for all gene families studied here, an appropriately selected subset of five or fewer regions covers the majority (>50%) of the event cost space (Fig. 3b), and coverage of the event cost space appears to follow a power law distribution (Supplementary Fig. S1). Although these results seem to suggest that a small set of event costs (and their associated regions) is representative of the entire event cost space, we note that coverage of event cost space is a biased measure, as some large regions may include cost ratios that are biologically unrealistic while other regions containing biologically plausible ratios might cover a small fraction of the event cost space. Regions of zero area, for example, are generally abundant and may contain a biologically relevant solution. While these results provide some preliminary understanding of the relationship of event costs and the space of maximum parsimony reconciliations, further research is needed to understand the properties of this space and, ultimately, to determine appropriate event costs.

Next, we focus on the identification of well-supported events across the event cost space. Given a user-specified event cost space, that is, a range of costs on transfer and loss (with respect to the unit cost of duplication), we used the *eventscape* tool to infer the Pareto-optimal regions and the sets of events that are common to 1,…,k regions, where *k* is the total number of regions in the event cost space. We define an event to have *consensus support s*, 0≤s≤1 (with respect to the event cost space), if the event is found in *every reconciliation* in at least a fraction *s* (inclusive) of the regions. Note that this is one of many possible measures of event support, and our algorithms can be used to define and compute other measures.

**Fig. 4.**
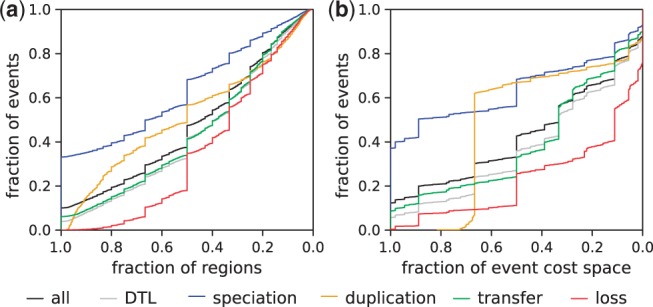
Event support for the tree of life dataset as measured by (**a**) fraction of regions or (**b**) fraction of event cost space covered. Coordinate (*x*, *y*) indicates that fraction *y* of events are found in at least fraction *x* of regions (or event cost space), with the plot being left-continuous (such that the highest *y* for each *x* should be read). Over all gene families, 16 795 speciations, 8375 duplications, 41 247 transfers and 13 761 losses are inferred

Using this definition, we investigated the fraction of events supported at various support thresholds (Fig. 4a). Almost universally, speciations are the best supported type of event, with ∼33.2% of speciations supported under strict consensus (that is, found in all regions). Manual inspection revealed that speciations found near the root of the tree were often conserved across multiple regions. However, when analyzing gene family evolution, we are typically interested in the inferred duplications, transfers and losses (collectively, the DTL events), and we found that few of these have high support. For example, only 2.1% of duplications, 15.1% of transfers and 2.1% of losses have at least 80% support (though the percentage of events with at least 50% support is substantially higher, with 56.8% of duplications, 41.4% of transfers and 34.9% of losses supported at that level). Interestingly, we observed a ‘jump’ in the number of supported events around a support threshold of 50%. Also, in general, for most support thresholds, duplications are the most supported type of event (after speciation), followed by transfers, then losses; however, for low support thresholds, the three types of DTL events show roughly equal support. These results have important implications for existing analyses that rely on DTL reconciliation and suggest that many inferred events are highly specific to the user-defined event costs.

In this study, we have investigated a specific range of event costs (transfer and loss ranging from 0.5 to 2 relative to the unit cost of duplication). The number of regions, and thus the level of consensus support, depends on this range. Moreover, our analyses permitted cost combinations in which duplications are more expensive or less expensive than transfers and losses. Adding constraints on these cost relationships could alter the levels of support. We also note that manual inspection revealed that loss events, and to a lesser extent transfers, are often ‘fungible’ in the sense that they can be moved from one location to another without changing the total cost of the solution, and thus any individual loss or transfer event is not likely to be strongly supported. By looking at the event cost space and supported events, our tools allow the elucidation of the complex interplay between event cost assignments and event support under various ranges and constraints of event costs.

For completeness, we also determined event support as measured by fraction of the event cost space (rather than fraction of the number of regions) covered (Fig. 4b), yielding similar results. The main difference between these two measures is that event support increases gradually with increasing region coverage but tends to ‘jump’ with increasing event cost space coverage; this confirms our finding that the event cost space is dominated by a few large regions. Otherwise, duplications also show a clear demarcation from no support to high support at ∼66.7% coverage.

To demonstrate the applicability of our tools on cophylogenetic datasets, we performed similar analyses on five host–parasite datasets and found results consistent with those above (Supplementary Section S3, Supplementary Fig. S2).

Finally, the average runtimes for our tools is on the order of seconds (Supplementary Table S1). To demonstrate scalability, we also ran *costscape* on the full 100-taxa tree of life dataset (also using transfer and loss costs ranging from 0.5 to 2) and found that the median runtime remains <1 min.

## 6 CONCLUSIONS

In this work, we have described new algorithms and tools for understanding the relationship between event costs and maximum parsimony reconciliations. In particular, we have given algorithms for computing the set of Pareto-optimal event count vectors and partitioning the event cost space into equivalence classes, or regions, induced by these vectors. We have demonstrated these algorithms in three software tools that (i) compute and visualize the regions that partition the event cost space, (ii) list the sets of events shared by reconciliations within and between regions and (iii) determine the statistical significance of reconciliation costs over the cost space.

An alternative approach to computing Pareto-optimal event count vectors would be to uniformly sample event costs and, for each sample, use existing algorithms to compute the maximum parsimony reconciliation and corresponding event counts. However, it is not known how to determine the appropriate sampling density to capture all event count vectors. Additionally, sampling fails to find regions that comprise points and lines, which we have shown to comprise a large fraction of the total number of solutions. Finally, our approach allows us to determine all Pareto-optimal event count vectors, whereas a sampling approach would necessarily capture only a subset of the event cost space.

Using the tools based on our algorithms, we have conducted experiments on a broad array of datasets. These results show that the space of maximum parsimony solutions is complex and sensitive to the event costs, and thus, choosing ad hoc event costs may result in misrepresenting evolutionary histories. At the same time, we cannot discount ad hoc procedures for finding event costs [for example, through comparison to event inferences on a biological dataset, as in David and Alm (2011)]. In particular, such approaches may elucidate the boundaries for biologically reasonable event cost assignments, which could then be used as input into our tools for more systematic analysis.

In addition, by defining notions of consensus support based on the number of regions that share an event, we found that while many speciation events have high consensus support, most other events do not. Thus, inferring events based on non-systematically selected event costs is likely to provide only a piece of a complex picture, and our tools motivate further investigation into the robustness of analyses based on DTL reconciliation.

There are numerous interesting directions for future work. In particular, this work affords opportunities to explore many variants of event consensus support. For example, our work uses the most specific definition of an event in computing support: for a speciation, duplication or transfer to be supported across different reconciliations, a gene (parasite) tree node must map to the same species (host) tree node and to the same event, and transfers must also yield the same transfer edge. Similarly, for two losses to be supported across different reconciliations, they must be found along the same branch of both the gene tree and species tree. In some cases, analyses only require the species mapping (David and Alm, 2011) or event mapping (Koonin, 2005), and thus the support values must be considered for these relaxed definitions of events. Our algorithms can be modified accordingly.

In addition to identifying highly supported events, it is desirable to find highly supported whole reconciliations. In the spirit of promising recent work on this problem for fixed event costs (Scornavacca *et al.*, 2013), one promising research direction is to identify and succinctly represent whole reconciliations that are robust across the space of event costs.

In summary, this work provides techniques and tools that are immediately useful in the phylogenomic, cophylogenetic and biogeography analyses and offers avenues for further research that leverages the Pareto-optimal reconciliation methods developed here
